# Atlanta Society of Medicine

**Published:** 1897-11

**Authors:** 


### SOCIETY REPORTS.

---

### ATLANTA SOCIETY OF MEDICINE.

ATLANTA, GA., September 21, 1897.

The meeting was called to order by the Vice-President, Dr. Jones.

The following members were present: Drs. Arch Avary, Benson, Gilbert, Hubbard, Hutchins, Dunbar Roy, Hood, Jones, Richardson, C. D. Hurt, Kime, Stockard, Gaston, Jr., Hancock, Robinson.

Mr. Davies, the attorney employed by the Society to prosecute illegal practitioners, was present and stated that his firm had employed men to get evidence against these practitioners, and that they now had all this evidence in shape. That they had at first sent out a circular letter to all on the list furnished to them, and that they had responses from all, with the exception of a few. That there were some of these irregular practitioners who would put up a hard fight, and that if his firm proceeded against them they would have to have the thorough support of the Society. That, according to the old law, all fines were payable to the prosecutor, but under the law of 1894 all these fines were payable to the State, and therefore the Society could not expect anything from fines to help pay the expenses of the cases. Therefore, he desired to receive instructions from the Society as to whether the cases should be pushed any further.

Dr. Hutchins moved that the Society instruct Mr. Davies to send out another letter to those who had not responded, requesting them to produce evidence of their qualifications to practice. Motion carried.

Dr. M. B. Hutchins described

### A PAINLESS METHOD OF VACCINATION.

"I have no paper prepared for to-night, but I propose to simply demonstrate a painless method of vaccination. Of course it is not painless after it has taken, but I thought I would like to get a new method for little children, and also for nervous people. We all have more or less trouble in vaccinating children, and I do not like the idea of scraping. The idea is simply this: The upper layer of the skin is a water-proof, or absorption-proof coating, and protects the derma and prevents absorption. Therefore, when we get rid of this epidermis, we have a surface which will absorb, and this is the principle of the whole thing. I thought of one or two things that would dissolve this epidermis without any pain. About two years ago I tried salicylic acid, and I have finally struck upon liquor potassæ. I have here a drawing of the skin, which will refresh your memory in regard to the anatomical structure, and you can see how the liquor potassæ acts. As soon as we remove this horny layer of the epidermis we have got down to the sensory filaments of the nerves, and also have here an absorbing surface. I have tried this method on myself several times, and there was simply a tingling, of irritation, and nothing more. By the old method we have a certain amount of pain, also some bleeding, which tends to prevent absorption, and then, too, it often scares the children, and makes it very troublesome in some cases.

"To vaccinate by this method you simply take a small piece of cotton and moisten it with the liquor potassæ, and place it upon the spot which you wish to denude. Allow it to remain upon the spot for a couple of minutes, or until a slight burning is felt, and sometimes you do not get this. You then take a pencil rubber, a piece of roller bandage, or anything of the kind, and rub off this epidermis, which has been dissolved by the liquor potassæ. The vaccine is then applied as in the other method.

"I think it is a much simpler and better method with children. My little girl vaccinated herself in this way without any trouble. This method, taken all together, is probably slower than the other, as you have to wait for the liquor potassæ to act. We could not well use the caustic potash, as it might burn too much. We have long used the liquor potassæ as a mounting fluid, and have seen how the cells melt under it and dissolve. When the liquor potassæ is put on the arm in this way it unites with the natural secretion of the skin and forms a soap. When I first put it on I have a slick, greasy surface which is hard to deal with. This combination is easily rubbed or washed off. The loss of time comes in after you put the cotton on, waiting for the liquor potassæ to get in its effect. But then perhaps it is not longer than it would take to get the child subdued under the old method.

"After using the potassa you simply rub off all the epidermis, and this will expose the papillæ of the skin. They are like the villi of the intestines and comprise the absorbing surface. After exposing these papillæ it will take on any person. I had a case sent to me the other day of a child who was very nervous, and she told me after I got through that it did not hurt her at all. I have used it on several students and it has worked admirably. I have applied the potassæ with a wooden tooth-pick to freckles and pigment surfaces of the skin, and in two or three minutes could take the tooth-pick and scrape off the pigmentation.

"Now, to show you the exact technique of this method, I will take this negro boy's arm and prepare it just as if I were going to vaccinate him. You take a small piece of cotton the size of the surface you wish to scarify and saturate it with the liquor potassæ and then apply it to the spot. You allow this to remain for a couple of minutes, or longer, until the patient feels a slight irritation at that point, and then you take a pencil rubber, or anything of the kind, and rub off all the epidermis. This discloses the papillæ of the skin and often their blood-vessels. This shows up beautifully on the negro, where you have the contrast of color. You then apply your virus to this place. There will be sufficient exudate to form a small scab. You can thus see that the method is very simple, and you do not have any bleeding. With sensitive people the idea of scraping and bleeding is very distasteful. I would like to have the candid opinion of the gentlemen in regard to this, as to whether it is something that will save trouble with small children and can be used to advantage.

"I have here a short report of some of the cases in which I have tried this method recently.

"Messrs. S., H. and W. were vaccinated on the 7th. Mr. S. had old scars and got no result. Mr. H. got no result and was revaccinated on the 20th. Mr. W. took well, two places, in six days.

"On the 8th Mr. H. No. 2 was vaccinated and was itching a great deal on the 10th, and within three days had twenty-five vesicles, mostly single, within the denuded area.

"Self; had good arm-to-arm vaccination fifteen years ago. Had abortive result in 1894 from the old method. On 8th of this month tried the painless denudation and had itching within twenty-four hours, which persisted nearly two days, and had three or four abortive lesions.

"Lady with an old scar. Vaccinated by denudation and had two characteristic lesions within ten days.

"Colored cook, who had never been vaccinated. One large denudation, and two points used. Took so well that she quit work for a week.

"Little girl of seven with faint old scar. Denudation and sterile rubber tissue dressing after using one point. In three days a vesicle, evidently taking. Later, this faded, was revaccinated, no report.

"Of seven cases never vaccinated, four took, and one doubtful, and two failed; one having failed previously by the old method. Of four previously vaccinated, showing old scar, one took, one had abortive lesion and two failed.

"Thus, allowing for poor virus, we have as good results as from the other method. The main point is that it is painless."

---

### THE COST OF THE YELLOW FEVER.

Although there has been comparatively little loss of life in yellow fever districts of the South, there has been a tremendous loss of money to the community because of the contagion. The New York *Herald* says it is estimated that the loss to the business men in New Orleans alone will amount to $25,000,000, while the losses in other directions and the cost of maintaining the quarantine will run the total up to $38,000,000. Most of this loss is the result of the wild and unreasonable excitement of the neighboring towns and villages that have shut off all supplies shipped from New Orleans and have absolutely paralyzed business in that city.—*Jour. Am. Med. Asso.*

